# Virtual reality intervention effects on future self-continuity and delayed reward preference in substance use disorder recovery: pilot study results

**DOI:** 10.1007/s44192-022-00022-1

**Published:** 2022-09-15

**Authors:** Yitong I. Shen, Andrew J. Nelson, Brandon G. Oberlin

**Affiliations:** 1 Department of Psychiatry, Indiana University School of Medicine, 355 W 16th St. Ste 4800, Indianapolis, IN 46202 USA; 2 Department of Psychology, Indiana University Purdue University Indianapolis, Indianapolis, IN USA; 3 Half Full Nelson, LLC, Indianapolis, IN USA; 4 Department of Neurology, Indiana University School of Medicine, Indianapolis, IN USA; 5 Stark Neurosciences Research Institute, Indianapolis, IN USA

## Abstract

Sustained remission from substance use disorder (SUD) is challenged by high relapse rates, which provides opportunities for novel clinical interventions. Immersive virtual reality (VR) permits delivering synthetic experiences that feel real and actualizes otherwise impossible scenarios for therapeutic benefit. We report on the feasibility of an immersive VR intervention designed to increase valuation of the future by enhancing future self-continuity and leveraging future self-discrepancy with personalized future selves as SUD recovery support. Twenty-one adults in early SUD recovery (< 1 year) interacted with versions of themselves age-progressed fifteen years from two different behavioral trajectories: an SUD Future Self and a Recovery Future Self. The future selves’ interactive monologs include personalized details and voice for a lifelike interaction within a time travel vignette. Before and following the intervention, participants rated future self-continuity and performed delay discounting. Following the intervention, daily images of the Recovery Future Self were sent to participants’ smartphones for thirty days. The VR intervention generated no adverse events, was well tolerated (presence, liking, and comfort), and significantly increased future self-continuity and delayed reward preference (doubling delay tolerance). The intervention also reduced craving, *p*s < 0.05. Thirty days later, *n* = 18 remained abstinent; importantly, increased future self-similarity persisted. Abstainers’ future self-similarity increased following VR. All individual participants showing increased future self-similarity post-VR remained abstinent, and all participants who relapsed showed either reduced or zero effect on future self-similarity. Post-intervention semi-structured interviews revealed emotional engagement with the experience. VR simulation of imagined realities reifies novel clinical interventions that are practicable and personalized. The current study demonstrates an implementation readily applied in the clinic and shows promise for facilitating SUD recovery. Creative collaboration between researchers, clinicians, and VR developers has great potential to revolutionize mental health interventions and expand the range of tools for clinicians targeting SUD and other disorders.

**Supplementary Information:**

The online version contains supplementary material available at 10.1007/s44192-022-00022-1.

## Introduction

“When you’re trying to help someone let go of a drug, you are competing with a powerful and long-practiced reinforcer. People change when they see an alternative that is better”.—William Miller, PhD [1].

Various substance use disorders (SUD) stubbornly resist remission—even with pharmacotherapy [2–4]; the majority of patients relapse within 6 months post-treatment [4–10], and 3-month rates can exceed 90% [4]. SUD behavior (excessive drug-taking and impaired physical, psychological, professional, and social well-being [11]) prioritizes immediate hedonic reward over future outcomes.

Undervaluation of future rewards—often quantified as the relative preference between an immediate reward and a larger delayed reward (temporal discounting)—is linked to SUD by a large body of literature (meta-analyses; [12–15]). These findings are potentially explained by shortened time horizons (time perspective), which manifest as an out-of-focus future that exerts minimal influence on current behavior [16–19]—dramatically illustrated by one study reporting healthy controls’ time perspective as 4.7 years and SUD as *9 days* [17]. This tendency is not readily explained by ignorance of contingencies, as awareness of immediate gratification costs does not necessarily predict advantageous choices [20]. In parallel with future time perspective, the future self-continuity model [21–24] posits that undervaluation of future rewards is explained by greater psychological distance between the present and future selves [25, 26]. Only the present self can experience stimuli and emit behavior, but the future self receives those positive or negative outcomes. In SUD, the present self enjoys drug reward and forces the future self to pay for it. While rooted in different perspectives, temporal discounting, time perspective, and future self-continuity make the same prediction for SUD decision-making: the perception of less salient and valuable future rewards biases preference toward immediate rewards. Therefore, increasing future self-continuity should increase delayed reward preference and focus on future consequences [21, 22].

Manipulations that increase sensitivity to future outcomes (meta-analyses; [27, 28]) are a compelling basis for emerging SUD interventions, with one promising method referencing specific future events (episodic future thinking; EFT [29–31]) to provoke mental simulation of the future [32]. EFT shifts time perspective [33] and is most effective when experiences are autobiographical [34], vivid [28], content-specific [35], episodic [32], future-oriented [33], and positive [28]. Using future selves to strengthen future thinking may have stronger ecological validity and is more generalizable, as versions of the self are expected to be present in *all* imagined self-relevant future events—potentially as the “feared self” or the “positive possible future self” [36]. Therefore, increasing future self-continuity should increase the relevance and salience of the future and delayed rewards [21, 23].

Immersive virtual reality (VR) is an accessible^1^ technology that vividly simulates otherwise impossible realities [37] and imparts “presence”—the illusion of actually being in the virtual place and experiencing the events [37–39]. VR’s use in addiction research mostly targets drug cue reactivity for studying craving, buts shows mixed results for reducing drug use [40, 41]. However, VR’s capacity for incorporating social and environmental elements into immersive experiences promises more robust and creative future paradigms [42]. VR also shows considerable promise in the flip side of addiction decision-making, i.e., increasing introspection and focus on healthy long-term goals. VR avatars of the self can promote prosocial behaviors, such as saving money [23], exercising [43], and behaving ethically [44]. Importantly, VR maximizes the vividness of future selves, which is key to increasing future self-connectedness [44, 45]. We reasoned that VR is particularly well-suited for targeting future outcomes in early recovering SUD. We developed a VR experience informed by schema theory [46] that incorporated key EFT elements portraying two personal futures in a VR vignette. We presented individualized age-progressed future selves to orient focus toward plausible futures: one unpleasant and one desirable. By extending theoretical conceptualizations of multiple possible future selves [36, 47, 48], we targeted future self-continuity [21, 22] and self-discrepancy [36, 49], i.e., dysphoria induced by actual versus ideal self dissonance—a key recovery change agent [50–53]. We recruited early-recovering SUD participants to experience a decision point and realistic expected future outcomes of SUD or sustained abstinence. After the VR intervention study day, we delivered 30 daily smartphone images of the recovery future self as visual retrieval cues.

We conducted an open-label study to evaluate a promising efficacy signal and the feasibility of using this novel VR intervention in a clinical context. Recent work indicates that VR methods would be adopted at high rates among SUD treatment professionals, particularly if VR demonstrated efficacy for increasing positive outcomes [54]. We hypothesized that the intervention would: (1) be well-tolerated, (2) increase future self-continuity, and (3) increase behavioral delayed reward preference. Exploratory analyses assessed relationships between the study day intervention response and recovery outcomes of interest at the 30-day follow-up.

## Methods

### Participants

Interested adults attending recovery programming in Indianapolis, Indiana, treatment centers and recovery houses for alcohol and/or substance use disorders completed a phone screen (including demographics, substance use history, treatment history, medical conditions, and current medications questions) to determine initial eligibility. Study inclusion criteria were: diagnosis and treatment for use disorders of alcohol and/or illicit drugs, early SUD recovery (< 1-year continuous abstinence,^2^ similar to NIAAA’s AUD remission definition [55]), greater than 14 days abstinent from drug/alcohol use at the time of the study day (exception: one enrollee with a single alcohol lapse six days prior to study), between ages 21 and 50, fluent in English, and actively engaging in recovery activities. Comorbid psychiatric conditions were permitted to maximize generalizability [56, 57]. Exclusion criteria included contraindications for VR or MRI (fMRI data to be reported separately), current use of alcohol or illicit drugs (including mu-opioid agonist pharmacotherapy), > 1 lapse event between the interview and the study day, disorders or history of neurological disease of cerebral origin, or head injury with > 20 min loss of consciousness. Participants agreed to participate in the study day and 30-day follow-up, with compensation for each phase completed ($250 total for full completion). All study procedures were approved by the Indiana University Institutional Review Board.

### VR

#### VR design and prototyping

Informed by prior work [23], we tailored a VR experience for early SUD recovery using age-progressed future selves. Our paradigm was carefully designed with consulting from long-term SUD-recovering members of the local community, treatment professionals, addiction psychiatrists, and faculty in the Indiana Alcohol Research Center. We crafted the VR experience to connect with the future in a narrative integrating established therapeutic elements: catharsis, new behaviors, and positive expectancies [58]. Feedback from volunteers and technical staff informed optimized artistic and technical aspects. Specific narrative elements were furnished by participants—reflecting their actual experience and/or expectations of continued SUD. We maximized presence, realism, and emotional impact [59] and optimized design features to avoid motion sickness [60, 61], discomfort [62], and uncanniness [63]. The design priorities of the intervention were: (i) personalization, (ii) a discrete decision point, (iii) instantiation of the future, (iv) contingency of present choice and future outcomes, and (v) agency. The 15-year aging interval was selected due to its effectiveness in teen participants [45], and displays sufficient visual markers of aging; this interval is intermediate to delays commonly used in temporal discounting tasks (e.g., [64]).

#### VR equipment

**Fig. 1:**
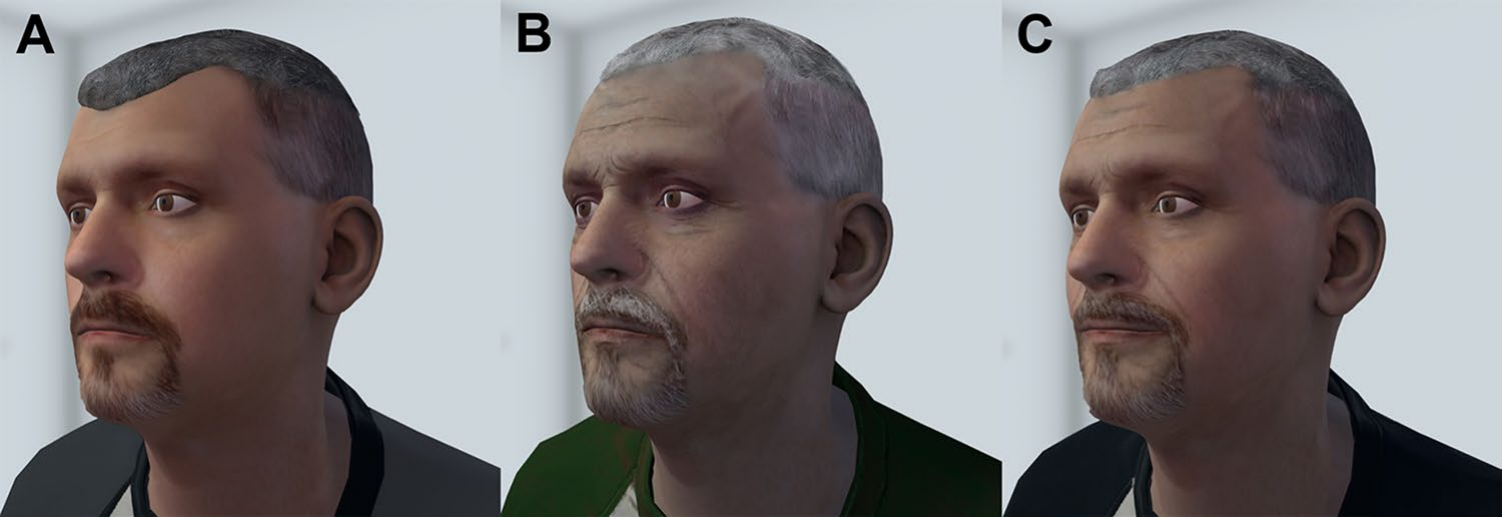
Avatar rendering. The senior author’s **A** Current Self avatar, and age-progressed 15 years **B** SUD Future Self, and **C** Recovery Future Self

All prototype pilot and test sessions were administered using a high-quality tethered headset and VR-capable computer. Photos and voice samples were utilized to construct personalized VR avatars; the Present Self, SUD Future Self, and Recovery Future Self (see the workflow as applied to the senior author, Fig. 1). The paradigm included a black steel park bench that was both real and digital, and co-registered in space; *Supplemental Materials* for details.

#### VR habituation

All participants experienced the virtual park for 4.55 ± 1.76 min (mean ± SD) to ensure non-novel VR. The city park was the same environment used for the intervention and included chirping birds, moving tree leaves, and lawn mowing. After a brief respite, participants experienced the VR intervention.

#### VR avatars

The Present Self avatars reflected participants’ current appearance. The age-progressed Future Self avatars were designed to appear 15 years older, with the SUD Future Self showing visible indicators of sustained drug use and the Recovery Future Self appearing healthy (*Supplemental Materials* for additional details).

#### VR intervention

Self avatars spoke to participants using a mostly fixed verbal script that matched their voice (manually-tuned text-to-speech engine) and incorporated important personal details provided in the structured interview. Social realism was maximized by avatars tracking participants’ position with their eyes and head movements. To maintain presence, technical staff remained silent and avoided touching participants aside from necessary physical guidance and instructions.

The paradigm progressed through four scenes and was designed to take 5 min (although participants’ behavior induced some variation with actual time taken being 5.7 ± 0.52 min). Scene 1 (white room): A narrator explained how VR permits simulated time travel. Participants established ‘body transfer’ [23, 65] in a virtual mirror. The Present Self then appeared as a full body, along with two park benches, and verbally recounted personal details. The Present Self foreshadowed multiple possible futures and presented a choice point, announcing, “choose your future.” Scene 2 (city park): Participants were teleported to the city park in the future and listened to the SUD Future Self describe the past 15 years and the ongoing costs of SUD. The SUD Future Self exhibited nonverbal and visual indicators of continued drug use. The participant was teleported back to the white room, where they ‘chose another future.’ Scene 3: (city park): Resembling Scene 2, but the healthy Recovery Future Self recounted the successes of the past 15 years. Scene 4: (white room): The Present Self debriefed the participant, promoted agency, and reiterated the temporal contingency between present behavior and future outcomes. See *Supplemental Materials* for further elaboration.

**Fig. 2:**
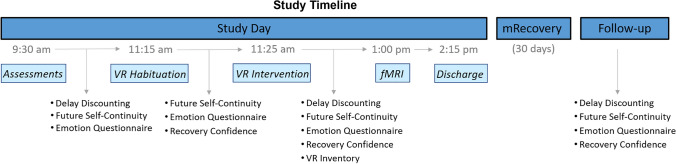
Study timeline. Discharge included Suicide Screening, Adverse Childhood Experiences questionnaire, and study day payment. Additional Assessments: Suicide Screening, Interpersonal Reactivity Index, Quality of Life Scale, Zimbardo Time Perspective Inventory, Sensitivity to Punishment and Sensitivity to Reward Questionnaire, Short UPPS-P Impulsive Behavior Scale, Aroma Choice task, and Stroop task (inventories and task order are randomized). mRecovery (‘Mobile Recovery’): daily telehealth text message (image of Recovery Future Self and Recovery Confidence item). Follow-up visit entailed urine drug screening, recovery-related outcomes, Quality of Life Scale, Suicide Screening, and payment for mRecovery

Participants completed delay discounting, the emotion questionnaire, and confidence in recovery item before and after the VR intervention, and the VR Inventory (Table S2) after the intervention. See Fig. 2 for timeline of study procedures. The ACE-Q was always collected after the intervention and behavioral tests to mitigate the potential effects of recalling childhood trauma. Immediately prior to discharge, participants were familiarized with the SMS messaging system (‘mRecovery’) with a smartphone test image and message (we planned to provide smartphones if needed, which proved unnecessary).

### Assessments

#### Demographics and biometrics

We collected demographic data, photographs, voice samples, and personal details (names of loved ones, favorite activities, future goals, substance use-related punishments, and preferred terminology for intoxication and abstinence).

#### Alcohol, drug use, and recovery

The Structured Clinical Interview for the Diagnostic and Statistical Manual for Mental Disorders Fifth Edition (SCID-5 [66]) yielded symptom counts for alcohol and the three most-used substances. The Timeline Follow-back (TLFB [67]) administered during the interview characterized participants’ substance use patterns 35 days prior to treatment. Accuracy was enhanced with memorable event dates, past text messages, calendars, pictures, and bank statements. NIAAA-defined heavy drinking (≥ 5 or ≥ 4 for males and females, respectively) days were identified, and illicit drug use was characterized by type and amount, in addition to current nicotine use. SUD family history was quantified by degree of relatedness with probable SUD positive relatives. We also characterized current recovery-related activities, treatment history, and confidence in remaining abstinent.

#### Personality, traits, and emotional states

Measurements included the Adverse Childhood Experiences Questionnaire assessing childhood trauma (ACE-Q [68]), recovery confidence (ad hoc self-efficacy item; ‘confidence in maintaining recovery’; 0–100 scale anchored by “not confident at all” to “very confident”; modified from [69]), the Interpersonal Reactivity Index for cognitive and emotional aspects of empathy (IRI [70]), emotion states questionnaire (modified from [23], see Table S3), the Zimbardo Time Perspective Inventory (ZTPI [71]), Sensitivity to Punishment and Sensitivity to Reward Questionnaire (SPSRQ [72]), trait impulsivity (short UPPS-P [73]), and Center for Epidemiologic Studies Depression Scale (CES-D [74]). Self-reported craving (“How much do I crave drugs right now?”) was assessed before and after the VR habituation and intervention, and at the follow-up, to monitor potential intervention effects. A brief suicide screen, Ask Suicide-Screening Questions (ASQ [75]), was conducted during the interview, before, and after the intervention to monitor participants’ safety. We administered the Quality of Life Scale (QOLS [76]) at the interview, on the study day, and at the follow-up to quantify participants’ self-appraised well-being (social life, personal development, and recreation).

#### Future self-continuity

The validated future self-continuity scale indexes the perceived psychological distance to the imagined future self [21, 22]. Participants endorsed future self-similarity and connectedness with Euler circle pairs (varying in overlap), labeled ‘Current Self’ and ‘Future Self.’ We used the original scale with two minor modifications: an additional option depicting complete overlap (suggested by Hershfield and colleagues [22]), Figure S1; and an expanded temporal window of 15 years to match the age progression of the future self avatars in the paradigm (the first four participants rated according to 10 years).

#### VR questionnaire

Participants rated the VR experience on elements of presence [77], tolerability/cybersickness, and enjoyment (detailed in Table S2).

### Behavioral and neuropsychological tasks

#### Delay discounting (DD)

An adjusting-amount DD task quantified relative preference for delayed rewards [78, 79]. Choices between smaller immediate versus larger delayed monetary rewards adjusted the next trial’s immediate amount down or up, respectively, allowing the procedure to converge on indifference points. The $100 amount was delayed by 2 days, 1 week, 1 month, 6 months, 1 year, and 5 years in 30 choice trials (5 trials × 6 delays). Participants were instructed to choose according to their real preference, and that some choices would be selected at random and paid according to their choice. The actual payout was an additional $20 at the end of the study day, obfuscated by computer selection and rounding.

#### Aroma choice task

The Aroma Choice Task (ACT) measures behavioral sensation seeking by quantifying the preference for intense, exciting, and novel sensory experiences [80, 81]. Participants chose between receiving a mild and pleasant odorant versus a more exciting (intense and varied aroma with an aversiveness risk) odorant [82, 83].

#### Stroop task

Executive attention was assessed using a 144-trial Stroop task [84].

### Study day procedures

On the study day, participants’ urine was screened for common illicit drugs (Wondfo Biotech, Ltd.), and exhaled breath was tested for alcohol (Alcotest^®^ by Dräger). Daily nicotine users were provided an appropriately-dosed nicotine patch (CVS Pharmacy, Inc.) to prevent withdrawal, unless refused (*n* = 2). Participants completed self-report inventories and behavioral tests interspersed with the VR paradigm;^3^ Fig. 2 for details.

### Telehealth; ‘mRecovery’ SMS

Participants received a scheduled Qualtrics SMS delivery for 30 days following the study day at randomized times between 10 AM and 10 PM. The SMS displayed an image of their Recovery Future Self on the park bench (VR screenshot). Beneath the image, participants rated “How confident am I that I will remain abstinent and recovering today?” on a visual analog scale from 0 to 100% (markers at 25% increments). The daily SMS push ensured image viewing and quantified confidence in remaining abstinent. We tested the system with participants at the end of the study day and clarified the incentives ($40 bonus for full completion with $2 omission penalties).

### Follow-up procedures

Participants completed follow-up procedures in-person 30 days post-study (actual follow-ups occurred at 38.57 ± 11.19 days; *n* = 2 required a phone follow-up). Procedures included self-reported alcohol/drug use, alcohol breath testing, and urine drug screening. Participants endorsing or testing positive for alcohol/drug use completed the TLFB to quantify use type, amount, and frequency. Participants completed the emotion states questionnaire, future self-continuity, QOLS, and DD task again.

### Power, analyses, and statistics

We estimated that *N* = 18 would be required to detect significant effects (G*Power 3.1.9.7 [85]), based on early work using future self avatars’ effect on temporal discounting [23], and the assumptions of alpha = 0.05 and power = 0.80. Paired *t*-tests assessed VR intervention effects on the study day (post- versus pre-VR) for future self-continuity, DD, emotional states, craving, and quality of life. Skewness and kurtosis are reported, and normality was assessed with Q-Q plots for the primary outcomes of interest (Figure S2); to mitigate against spurious effects due to non-normality or outliers we also assessed natural log normalized values (post-VR minus pre-VR, with 2 added to generate positive values, and one-sample *t*-tested against 0.6931 [i.e., *ln*2]). Exploratory analyses tested for differences at the 30-day follow-up to assess the persistence of those effects. Exploratory analyses also examined group differences between those who remained abstinent (*n* = 18) versus those who relapsed within 30 days (*n* = 3) in demographic variables, outpatient recovery activities, future self-continuity change, and short UPPS-P scores, with additional assessment of executive function and impulsivity traits. Demographic factors with potential explanatory interest (Table 1) were tested for effects of group (*t*-test or Chi-Square). Spearman correlation tested for associations between the response to the intervention on the study day, follow-up day (delta future self-continuity and DD), and other factors of interest. Change in use was tested against zero in a one-sample *t*-test in those who relapsed. The alpha level was set to 0.05 (two-sided) for all results, with effect sizes reported as Cohen’s *d* and in-text values as mean ± SD unless otherwise specified. Primary analyses were pre- versus post-VR, uncorrected for multiple testing due to the feasibility nature of the study. Analyses were performed with SPSS v28 (Chicago, IL).

**Table 1:** Participant characteristics

Characteristic	Total (*N* = 21) Mean (SD) or *n* (%)
Age	34 (7.9)
Sex (male)	15 (71.4)
Race (white)	19 (90.5)
Years of education	14.9 (3.1)
Days of abstinence	74.4 (44.7)
Current nicotine use	14 (66.7)
Family history AUD^a^	11 (52.4)
Family history SUD^a^	9 (42.9)
Recovery activities per month^b^	23.7 (15.5)

^a^At least one 1st degree relative with use disorder

^b^Appointments and/or group meetings

## Results

### Participants

Phone screens (*n* = 189) and disqualifications (in descending order: MRI contraindications, current psychotropic medications, length of abstinence, age, health conditions, and VR contraindications) yielded 91 participants scheduled for in-person interviews. We obtained informed consent from 34 participants, with 13 of those later disqualified (MRI/VR safety concerns, VR technical difficulty, lost contact, positive breath reading/drug screen or no-show on the study day, major medical history concern, or > 1-year abstinence). The final *n* = 21 sample is summarized in Table 1. Alcohol and polysubstance use were highly prevalent, with AUD criteria met for *n* = 19 (90%) and use disorders of more than one substance met for *n* = 17 (85%); see Table S1.

### Retention

Of *n* = 21 study day completers, retention was 100% (although phone follow-ups were required in *n* = 2).

### Adverse events

There were no expected or unexpected adverse events observed in this study.

### VR tolerability, usability, and emotion states

Average score of presence subscales was moderately high (4.7 ± 1.4 on a 1–7 scale, anchored by “Not at all” to “Very much”), with good tolerability (1.1 ± 0.1 on a 1–4 scale, anchored by “None” to “Severe”), high liking and comfort (6.4 ± 0.7 on a 1–7 scale, anchored by “Strongly Disagree” to “Strongly Agree”), and not too heavy (rating of 1.8 ± 0.9 on a 1–7 scale, anchored by “Strongly Disagree” to “Strongly Agree”). Table S2 details all items and scores. The VR experience did not change negative (1.7 ± 0.9 and 1.6 ± 0.7, post- versus pre-VR, respectively) or positive emotions (5.2 ± 1.3 and 5.3 ± 1.1, post- versus pre-VR, respectively), *p*s > 0.5. See Table S3 for all items and scores.

### Suicide screening

The ASQ was performed pre- and post-VR intervention on the study day and at the follow-up. No participants reported suicidal thoughts during the past month. Five participants reported making a suicide attempt in the past (all were > 5 years prior to study participation). No changes in suicidal thoughts or attempts were reported. Note: the first participant was not screened, and another omitted the ASQ, given remotely (due to Covid-19).

### VR effects on the imagined future self and delayed rewards

#### Future self-continuity

**Fig. 3:**
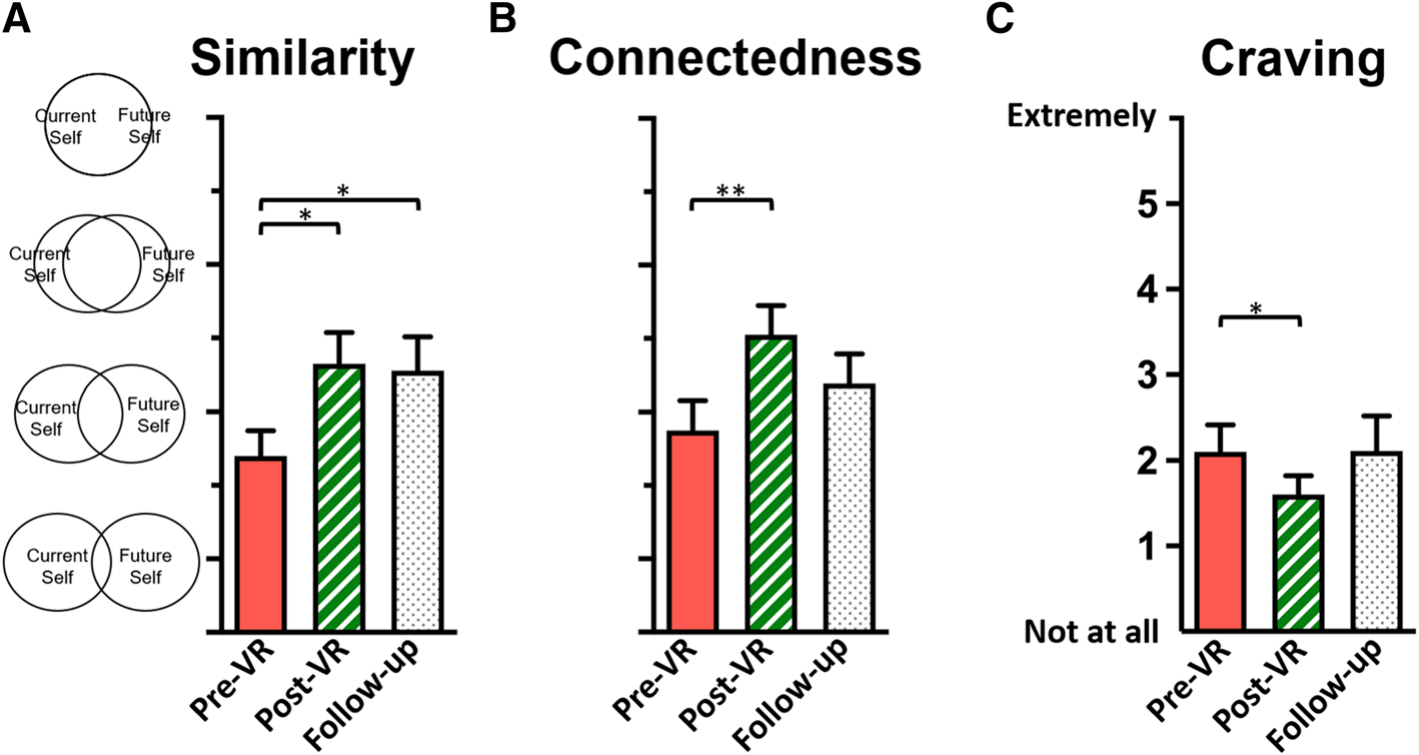
Intervention effects. Increased future self-continuity was detected following VR in **A** future self-similarity, on the study day and persisting 30 days later, and in **B** future self-connectedness. **C** Craving decreased following the intervention; *ps < .05, **p < .001

The VR intervention increased participants’ future self-similarity post versus pre-VR (1 missing datum for pre-VR), *t*(19) = 2.80, *p* = 0.011 (Fig. 3A), skew = 1.60 and kurtosis = 2.73 (log-normalized: *t*(19) = 2.62, *p* = 0.017, skew = 0.16 and kurtosis = 0.13), and future self-connectedness post versus pre-VR, *t*(19) = 5.38, *p* < 0.001 (Fig. 3B), skew = 1.00 and kurtosis = 0.80 (log-normalized: *t*(19) = 6.47, *p* < 0.001, skew = 0.26 and kurtosis =  − 0.20).

#### Delay discounting

**Fig. 4:**
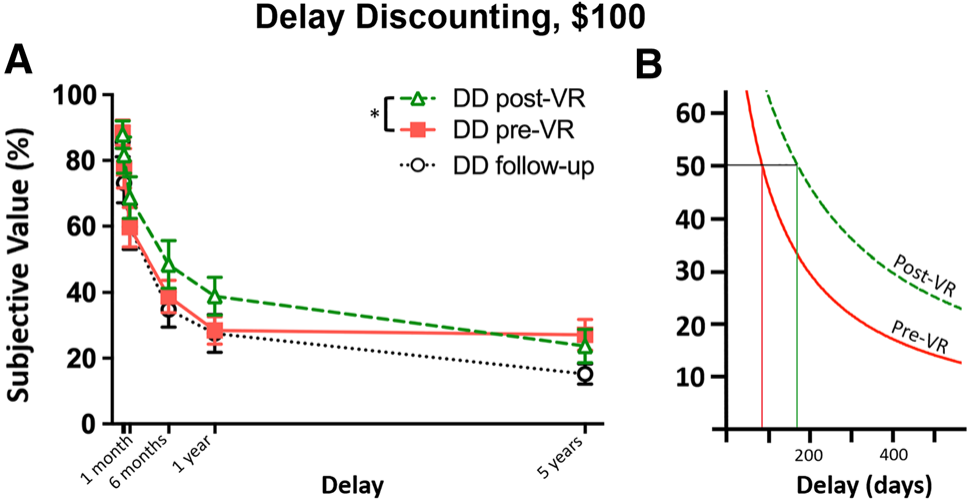
Intertemporal choice. **A** VR reduced delay discounting for monetary rewards on the study day; effects were not detected 30 days later; *p < .05. *Note: delays of 2 and 7 days were omitted from the x-axis for clarity.* **B** Nonlinear (hyperbolic) curve fits illustrating increased delay tolerance, quantified as the delay corresponding to the halving of subjective value of the delayed reward (ED_50_; pre-VR = 85.8 days, post-VR = 170.9 days)

The VR intervention increased delayed reward preference in the DD task (area under the curve) post- versus pre-VR (0.36 ± 0.24, and 0.26 ± 0.14, respectively), *t*(20) = 2.36, *p* = 0.029, skew = 1.79 and kurtosis = 3.72 (log-normalized: *t*(20) = 2.38, *p* = 0.028, skew = 1.54 kurtosis = 2.88). To quantify delay tolerance in units of time delay, *k* (the fitted parameter indexing discounting) was used to calculate ED_50_—the delay corresponding to the halving of subjective value (1/k [86])—which revealed a doubling of delay tolerance for rewards, post- versus pre-VR; Fig. 4.

### Exploratory analyses

#### Abstinence and recovery activities

The VR intervention yielded alcohol/drug abstinence in *n* = 18 (86%) 30 days following the study day. The post-study time to relapse in the *n* = 3 was 5.3 ± 7.5 days. The total amount of drug use was reduced at follow-up compared to prior-to-treatment use amount reported at the interview, *t*(2) = 8.76, *p* = 0.013. Qualitatively, those *n* = 3 who relapsed (#1) refrained from their primary drug of choice entirely (alcohol), and the quantity of marijuana used dropped 71.6%; (#2) methamphetamine use declined by 68.6%; and (#3) use dropped by 97.6% and 97.0% for alcohol and cocaine, respectively. Participants remaining abstinent did not differ in demographic characteristics (Table 1) from those who relapsed (*p*s > 0.1), except that those relapsing had more years of education than abstainers (18.7 ± 0.6 and 14.3 ± 2.9, respectively, *t*(19) = 2.59, *p* = 0.018).

#### Future self-continuity response, abstinence, and self-efficacy

**Fig. 5:**
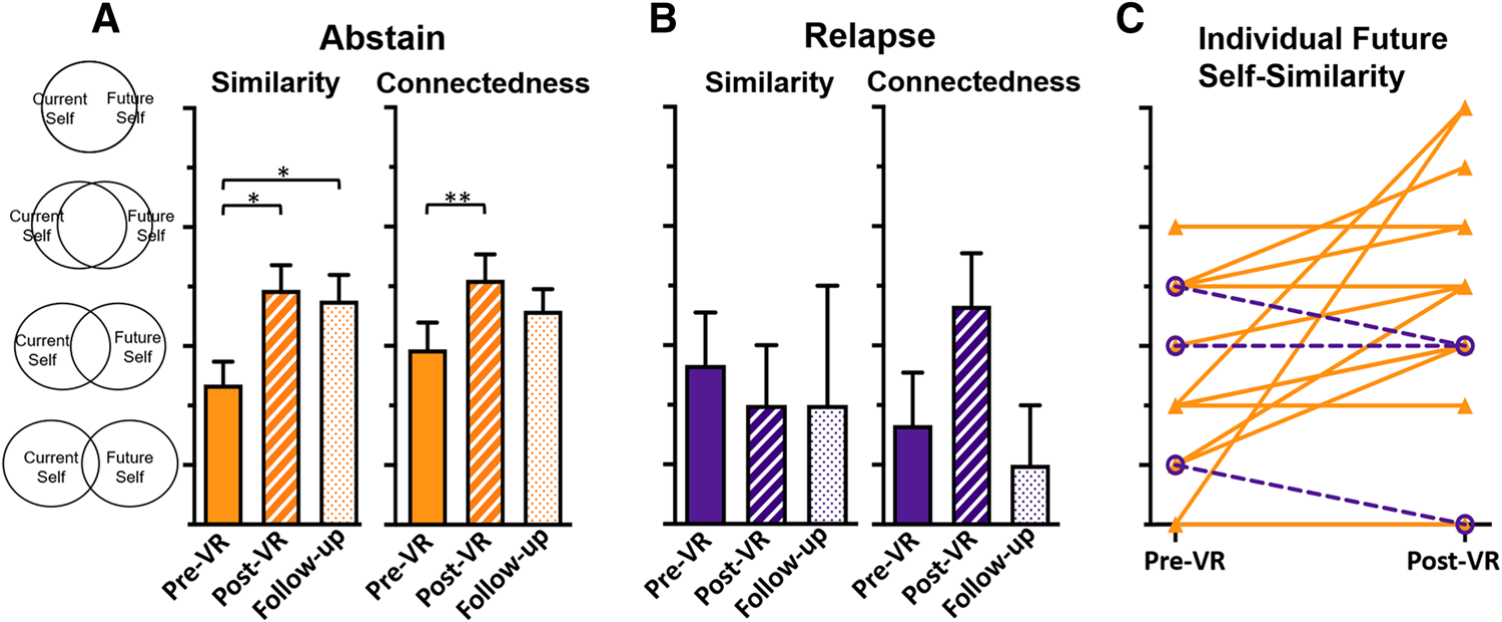
Future self-continuity. **A** In abstainers, future self-similarity (left) and connectedness (right), and **B** in those who relapsed. **C** Individual responses to VR in abstainers (closed triangles, solid orange) or those who relapsed (open circles, dashed purple). *ps < .05, **p < .001

We examined intervention response to potentially explain relapse. The VR experience increased future self-similarity in abstainers post- compared to pre-VR [*n* = 17 (one missing), *t*(16) = 3.32, *p* = 0.004] but not in those relapsing (*n* = 3, *p* > 0.1); Fig. 5A, B. The change (delta) in post- versus pre-VR future self-similarity between abstainers and those relapsing showed a trend-level difference (1.6 ± 2.0 and − 0.7 ± 0.6, respectively), *t*(18) = 1.93, *p* = 0.070, but did not reach significance. Importantly, all participants showing a positive response in future self-similarity (an increase post- versus pre-VR intervention) remained abstinent. Those who relapsed showed either a negative or no change (Fig. 5C).

#### Sensation seeking and impulsivity

Participants reported representative Sensation Seeking scores relative to published means from an SUD population [87]; 11.3 ± 3.2 versus 11.1 ± 2.9, respectively, *p* > 0.7. Comparing those who remained abstinent to those who relapsed for trait differences revealed that abstainers were higher sensation seekers than those relapsing (11.9 ± 3.0 and 7.7 ± 2.5, respectively); *t*(19) = 2.32, *p* = 0.032, but did not differ in other subscales of UPPS impulsivity (*p*s > 0.2). Behavioral sensation seeking (measured by ACT) did not correlate with delta DD or delta future self-continuity (post- versus pre-VR and follow-up versus pre-VR), *p*s > 0.2. ACT choice ratio did not differ between abstainers and those who relapsed, *p* > 0.4.

#### Craving

The VR intervention significantly reduced participants’ self-reported craving post versus pre-VR, *t*(19) = 2.36, *p* = 0.029, although this appeared to be a transient effect that waned 30 days post-VR; Fig. 3C.

#### Empathy

Personal Distress negatively correlated with the change in future self-similarity at follow-up compared to baseline, *r*_s_(17) = − 0.51, *p* = 0.027. The Empathic Concern subscale correlated positively with the change in post- versus pre-VR intervention in future self-connectedness, *r*_s_(18) = 0.57, *p* = 0.009 and negatively with future self-similarity, *r*_s_(18) = − 0.44, *p* = 0.0496.

#### 30-day follow-up

Change in future self-similarity post- compared to pre-VR correlated with self-reported ratings of recovery importance at follow-up, *r*_s_(16) = 0.51, *p* = 0.031, and confidence in recovery at follow-up *r*_s_ (16) = 0.55, *p* = 0.017. Participants’ future self-similarity was significantly higher at follow-up compared to pre-VR (4.6 ± 1.9 and 3.4 ± 1.5, respectively), *t*(17) = 2.29, *p* = 0.035. Abstainers’ future self-similarity response on the study day persisted for 30 days (follow-up versus pre-VR, 4.8 ± 1.8 and 3.4 ± 1.6, respectively), *t*(15) = 2.36, *p* = 0.033 (one missing datum). Participants’ quality of life (measured by QOLS) significantly improved at the follow-up relative to the study day (80.1 ± 11.7 and 76.5 ± 15.0, respectively), *t*(15) = 2.37, *p* = 0.032. The effect of the VR intervention on DD, while significant on the study day, did not persist at follow-up (*p* > 0.8, relative to pre-VR DD).

#### VR history, pre-habituation VR comparison, and intervention response

Eight participants (38%) had previously experienced VR in some form. The VR habituation did not significantly change future self-continuity (connectedness and similarity, *p*s > 0.4). It did reduce negative mood, however (post-habituation versus pre-habituation average subscale scores 1.71 ± 0.94 and 1.61 ± 0.66, respectively), *t*(19) = 3.88, *p* = 0.001. Participants with or without prior VR experience did not differ in pre-VR future self-connectedness, pre- or post-VR DD AUC, change in future self-similarity post- versus pre-VR, change in future self-similarity at follow-up compared to pre-VR, change in DD AUC post- compared to pre-VR, or change in DD AUC follow-up compared to pre-VR (*p*s > 0.1). However, pre-VR future self-similarity was significantly higher in those with prior VR experience compared to those without (4.7 ± 1.3 and 2.7 ± 1.2, respectively), *t*(18) = 3.58, *p* = 0.002.

### Executive attention (Stroop task)

Executive attention negatively correlated with delta future self-similarity post- versus pre-VR, r_s_(15) = − 0.49, *p* = 0.044, but not delta future self-similarity follow-up versus pre-VR or delta future self-connectedness (*p*s > 0.05). Interestingly, executive attention positively correlated with delta DD follow-up versus pre-VR, r(14) = 0.52, *p* = 0.041, but not post- versus pre-VR (*p* > 0.7). Executive function in abstainers and those who relapsed did not differ, *p* > 0.6. One outlier Stroop score was excluded (3.66 SD below the mean).

#### Subjective feedback

We initiated sound recording during post-study day interviews after the study commenced. For the *n* = 12 recorded, all reported that the VR intervention was memorable. Eleven recounted that it was emotional and helpful, and 10 described it as believable and affirmed the technical realism. Ten desired an extended-length VR experience. See Table S4 for representative comments.

## Discussion

To our knowledge, this is the first intervention designed to provide SUD recovery support using age-progressed avatars in VR, targeting future self-continuity and temporal discounting. By experiencing vivid and realistic personal futures, participants showed greater future self-continuity (which persisted 30 days later), increased delayed reward preference, reduced craving, and refrained from drug use at high rates. Importantly, the effect on future self-similarity tracked with abstinence, with increased future self-similarity in abstainers, and a lack of response with relapse. Interestingly, abstainers were higher sensation seekers than those relapsing, raising the possibility that this intervention may interact with a predilection for high-intensity experiences. The intervention was well-tolerated and demonstrated the feasibility of implementing a VR intervention in the clinic.

SUD can be conceptualized as overvalued immediate drug reward and undervalued (imagined) longer-term rewards [88]. Inverting this preference should facilitate SUD remission. While cue reactivity and craving are attractive targets for attenuation, more future development is required for effective VR cue exposure therapies (CET) [89]. While CET efficacy may be ultimately limited by the persistence of associative learning that cannot be truly unlearned [90, 91], the power of yet-untested new VR experiences may extend those boundaries [42]. VR is well-suited for creating potentially profound experiences that are autobiographical, vivid, immersive, emotional, and interactive. Therefore, increasing the value of future rewards through attentional and executive focus should shift preference toward more adaptive prosocial delayed reward choices. This massive shift in reward-seeking strategy, reorientating reward drive from immediate hedonic drug rewards toward imagined long-term healthy rewards, potentially reflects the quantum change [92], variously described as a “moment of clarity” [93],^4^ or metaphysically as a “spiritual awakening” (e.g., Step 12 [94])—an often pivotal process for SUD recovery [92, 95, 96].

Autonoetic (self-knowing) consciousness permits humans to mentally simulate themselves in different scenarios in time and space[97, 98].^5^ ‘Mental time travel’—projecting oneself into specific imagined personal events—is believed to increase the subjective value of future rewards [32, 99]. We created a VR intervention that incorporated key future thinking elements (autobiographical, future-oriented, vivid, content-specific, and positive [28, 100]), making distant, abstract future rewards potentially capable of competing with drug reward for future-myopic SUD. From an autonoetic perspective, steeply discounting the future arises from pleasing the present self by robbing the future self—an obvious analogy to SUD behavior.

We detected positive correlations between the intervention-induced increase in future self-similarity and both confidence in recovery and recovery importance. These findings suggest an intriguing possibility—that the capacity to increase future self-similarity corresponds to recovery self-efficacy-related traits. The questions we posed addressed “recovery” as a superordinate construct encompassing self-identified recovery-related behavior, rather than abstinence. Insofar as recovery confidence and importance map onto abstinence self-efficacy, this may be an important relationship. Abstinence self-efficacy is key for SUD behavior change, with high confidence in post-treatment abstinence the single strongest predictor of 1-year abstinence [101]; of note, abstinence self-efficacy is also predicted by delay discounting rates in recovering SUD [102]. Executive attention may be more closely aligned with intertemporal choice rather than future self-similarity, as we detected divergent associations with VR changes in these measures.

We conceived the fictional time travel storyline around two central themes: a decision point with agency and salient visible future outcomes. The narrative featured personally-imagined outcomes presented in familiar terminology to maximize engagement and persuasive impact [103]. While the experience could potentially be constructed to present just one future self, we preserved the decision-point-to-outcome contingency by illustrating two plausible outcomes. By animating both selves, we pit the “feared self” against the “positive possible future self” to elicit motivational power [36]. Prior work using therapeutic VR experiences shows that both negative and positive versions of the self possess motivational efficacy [43]. Future iterations of the current intervention should ascertain if similar effects could be obtained with only one future self.

VR applications targeting SUD require special design considerations beyond the standard technical concerns related to simulation sickness [60], namely attention toward exacerbating craving states [104] or comorbid psychiatric conditions (particularly anxiety disorders) or symptoms of trauma (for review, [105]). Informed consent documents must clearly outline the nature of VR, especially for the uninitiated, due to the “superrealism” of modern VR [106]. The nascent field of VR telehealth poses new risks regarding confidentiality [105], which must be carefully managed. We measured craving and suicidality in particular to assess potential psychiatric risks our paradigm posed for this subject population, and were encouraged to find *decreased* craving and unchanged suicidality (while craving was not a primary outcome, it was an important marker for potential risks of the intervention). Comporting with modern clinical applications of VR, our participants tolerated the VR hardware well (high comfort, low negative effects) and found the experience enjoyable. Despite the realistic portrayal of a decidedly negative version of themselves, participants found the overall experience positive. There were no adverse events observed during the course of the study. Two participants’ comments in particular well-articulated the intent of the intervention: “*If I stay on the path, I can see the life I envisioned for myself*” and “*It made me imagine what it would be like the next 15 years to get to that point, all the wasted money, opportunities, and relationships—it made me feel sad. Hearing myself saying getting sober was the best decision I ever made stuck with me.*” The non-directive feedback highlighted a major strength of VR; even though the experience is not real, the cognitive and emotional states it elicits *are* real. We believe that carefully designed therapeutic VR experiences have the potential to facilitate emotional epiphanies and the progression to the action and maintenance stages of change. Our results suggest that interacting with virtual age-progressed future selves increases attention on the future: a feature that is often invoked clinically, but difficult to convincingly present in a therapeutic setting. It is easy to imagine modified extensions of the current paradigm to other psychiatric and psychological conditions requiring greater attention on the future. Moreover, with the advent of inexpensive standalone headsets and user-friendly software, therapeutic VR is increasingly accessible to clinicians and practicable for interventions.

Several limitations should be considered. First, this feasibility study was limited in sample size and lacked a control group. While the lack of control groups does not preclude claims of within-subject differences pre- and post-VR, effects related to demand characteristics cannot be ruled out. Our primary goal was to establish that an intervention utilizing digital future selves in immersive VR could produce meaningful changes in future-oriented outcomes (future self-continuity and temporal discounting). Future iterations will employ controls for a simple VR experience and VR interaction with self avatars. Second, future self-continuity was somewhat open to interpretation, raising the possibility that participants did not all visualize the same future self when rating similarity and connectedness. We “deliberately left the concept of similarity open to interpretation for our research participants” (Hershfield 2011, pg. 6 [21]), presuming that the singular imagined future self represented participants’ exemplar of their personalized futures. Third, while we described ‘mRecovery’ as an assessment, it is arguably also an intervention. While our analyses focused on the primary outcomes assessed on the study day (unaffected by ‘mRecovery’), our exploratory analyses included effects potentially influenced by ‘mRecovery’. Indeed, telehealth SUD interventions may rival in-person therapy [107] and warrant further testing for additive effects in future studies. Finally, although the effects on temporal discounting did not persist 30 days later, the intervention demonstrated short-term efficacy, suggesting promise for future iterations presenting repeated future self experiences that may produce more durable effects.

We believe this approach shows considerable promise for much-needed interventions promoting SUD remission. These findings provide the basis for future randomized controlled trials using larger samples and potentially higher “doses” of the intervention. Future investigations should optimize parameters and identify the “active ingredients” to maximize effects. Accelerated VR adoption suggests that the potential for VR to be used in clinical settings will continue to expand, providing increasingly sophisticated tools for clinicians.

## Supplementary Information

Below is the link to the electronic supplementary material.

Supplementary file1 (DOCX 16 KB)

Supplementary file2 Figure S1. Future Self-Continuity Scale. The degree of overlap indicates how “Similar” and “Connected” one feels to the 15-year older future self. (TIF 2092 KB)

Supplementary file3 Figure S2. Q-Q Plots. (A) Primary outcomes (post-VR minus pre-VR) and (B) natural-log transformed (constant-added positive values) are shown with line of identity (dotted). (TIF 3456 KB)

Supplementary file4 (DOCX 29 KB)

## Data Availability

The datasets generated during and/or analyzed during the current study are available from the corresponding author on reasonable request.

## Footnotes

1 High-performance standalone (untethered) VR headsets are widely available in the global marketplace and cost ~ $300 in the US in early 2022 (e.g., Oculus Quest™).

2 We defaulted to the definition commonly accepted among our participants and in our partner treatment centers. Out of 18 participants queried on their personal definition of recovery-compliant drug use, 15 participants endorsed complete abstinence from alcohol and all illicit drugs, and three participants allowed for some future use under particular conditions: use of marijuana if legalized, occasional drinking, and hallucinogens following one year’s abstinence.

3 Participants underwent MRI testing following VR and behavioral tasks; those procedures and data will be reported elsewhere.

4 A common thread running through these 44 autobiographical stories of struggles with addiction and recovery is an emotional epiphany coinciding with a dramatic re-ordering of reward-seeking priorities.

5 Autonoetic consciousness is a specific brain function that permits episodic future thinking. Tulving [97] offers a telling account of a man who lost this ability, through traumatic brain injury, yet retained largely intact language, general knowledge, and understanding of chronological time. Unable to imagine himself in the future, patient N.N. could not describe what he will do tomorrow. When making these attempts, he characterizes his state of mind as a “blankness”; “It’s like swimming in the middle of a lake. There’s nothing there to hold you up or do anything with.” (p. 4). See Klein et al. [98] for a similar example of hypoxia-induced selective episodic dysfunction with intact semantic performance.
