# Supplementary material for: Virtual reality intervention effects on future self-continuity and delayed reward preference in substance use disorder recovery: pilot study results
Source: Discov Ment Health. 2022 Sep 15;2(1):19. doi: 10.1007/s44192-022-00022-1 (PMC9477176; doi:10.1007/s44192-022-00022-1)
Supplement: Supplementary file 1 — Supplementary file1 (DOCX 16 KB) [file 44192_2022_22_MOESM1_ESM.docx]

**VR equipment specifications.** All prototype pilot and test sessions were administered using the Samsung Odyssey™ head-mounted display with 6 degrees of freedom (Single Fresnel, 110° lens, AMOLED 3.5” 1440×1600 resolution at 90Hz per eye) on an Acer Predator™ laptop (Predator 15, model N15P3 with Intel^®^ Core™ i7-7700HQ CPU, 16 GB RAM and NVIDIA® GeForce GTX™ 1070 graphics card) and Microsoft Windows 10 operating system. Prototype and pilot testing were conducted in the senior author’s sound-attenuated basement laboratory studio. High-resolution photos of the head (facing, 45°, and profile at 1.1 meters, and macro of eyes) and full body (facing, profile, and T-pose at 2.8 meters) were taken with a Sony camera (Sony ZEISS Vario-Tessar, 20.1MP effective resolution). A 30-second voice sample (participants’ description of their morning) was recorded on a Dell Latitude 3500 data collection laptop. Our VR developer partners used the visual and audio data in a custom workflow to construct and animate the personalized avatars. Participants sat on a black steel park bench (dimensions 119×46×76 cm), which was precisely modeled as a 3D object and matched in spatial position within the virtual park. To enhance sensory engagement with the paradigm, participants were immersed in the scent of cut grass (New Mown Grass odor; International Flavors & Fragrances Inc., New Jersey) using an in-house custom scent diffusor (4 odorant-saturated polymer pellets diffused with a battery-powered brushless 12v fan) mounted to the underside of the bench. The diffusor was activated during the park scenes, which included a distant animated lawn-mowing worker and a background lawnmower sound.

**VR avatars.** The Present Self avatar was designed to look identical to the participant’s present appearance. The future self avatars were age-progressed 15 years using a customized workflow, with some hand editing to maximize realism for modified hairlines, graying, and wrinkling. The diminished physical and psychological states of the SUD Future Self were instantiated with additional aging and indicators of poor self-care (darkening around eyes, color desaturation in the skin, and unkempt clothes). Animation of the future selves reinforced nonverbal narrative elements of the paradigm: the SUD Future Self was unsmiling, downward gazing, slumped in posture, and more fidgety. In contrast, the Recovery Future Self smiled slightly while speaking, maintained eye contact and upright posture, and appeared calm and poised.

**VR paradigm.** The written script was customized using specific names, events, and vernacular provided by the participant and was translated to audio using Google’s text-to-speech engine (matched to participants’ sex and age, and manually tuned using the voice sample to approximate pitch). The realism of the avatars’ verbal delivery was maximized with a combination of Autodesk Maya and lip-sync animation tools in the Unity game engine to precisely register phonemic mouth, lips, and facial musculature to spoken words.

**VR intervention.** The paradigm progressed through four scenes. Scene 1: Participants were introduced to the paradigm by standing and viewing a large bare white room. A disembodied narrator (voice actor recording) described virtual reality as a place not limited by time and space, and anything was possible. The narrator instructed the participant to turn around, look into ‘the mirror,’ and move around (to establish ‘body transfer’). The Present Self avatar’s position linkage created the appearance of reflected head movements in the head-high virtual mirror. Mirrored movement continued for 24.6±8.8 seconds, after which the mirror and wall dissolved in a flash of white light, which revealed the entire body of the Present Self and two nearby black park benches oriented at 90°, one meter apart. The Present Self gestured toward one bench and invited the participant to sit down, after which the Present Self sat on the other. The Present Self reinforced identification by naming loved ones, birth year, favorite activities, motivations for recovery, and foreshadowed the experience by highlighting the participants’ age at 15 years in the future. The Present Self then produced two large crystal balls (hued green and blue) and asked participants to “choose a future” and time travel 15 years into the future. Looking at a ball for two seconds initiated a white light and scene transfer to the park. Scene 2: The SUD Future Self avatar sat adjacent to the participant on the other bench. Two seconds of eye contact initiated the monolog, wherein the SUD Future Self recounted negative outcomes of continued drug use (obtained from the interview) and missed opportunities for recovery. A white light flash teleported the participant back to the white room with the Present Self for a scene change. Scene 3: The Present Self invited the participant to “choose another future,” with the same transition as Scene 2. Teleported back to the park, the participant viewed the Recovery Future Self sitting on the adjacent bench. Sitting closer to the participant, the Recovery Future Self described a successful recovery journey, referencing the participant’s anticipated positive future outcomes (obtained from the interview). The Recovery Future Self then affirmed the participant and praised them for their hard work. Scene 4: Returning to the white room, the Present Self encouraged the participant, reinforced positive agency, and restated the contingency between the participant’s present actions and future outcomes.
