# Supplementary figures and images for: Virtual reality intervention effects on future self-continuity and delayed reward preference in substance use disorder recovery: pilot study results
Source: Discov Ment Health. 2022 Sep 15;2(1):19. doi: 10.1007/s44192-022-00022-1 (PMC9477176; doi:10.1007/s44192-022-00022-1)

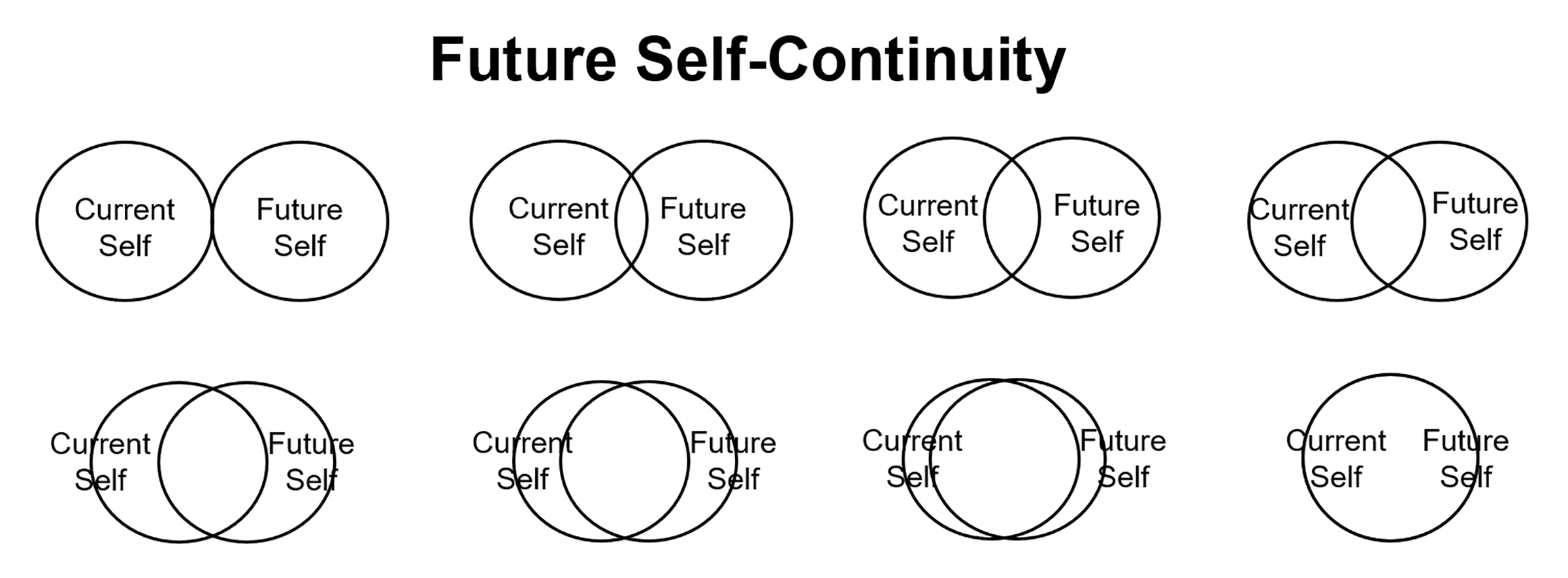

Supplement: Supplementary file 2 — Supplementary file2 Figure S1. Future Self-Continuity Scale. The degree of overlap indicates how “Similar” and “Connected” one feels to the 15-year older future self. (TIF 2092 KB) [file 44192_2022_22_MOESM2_ESM.tif]

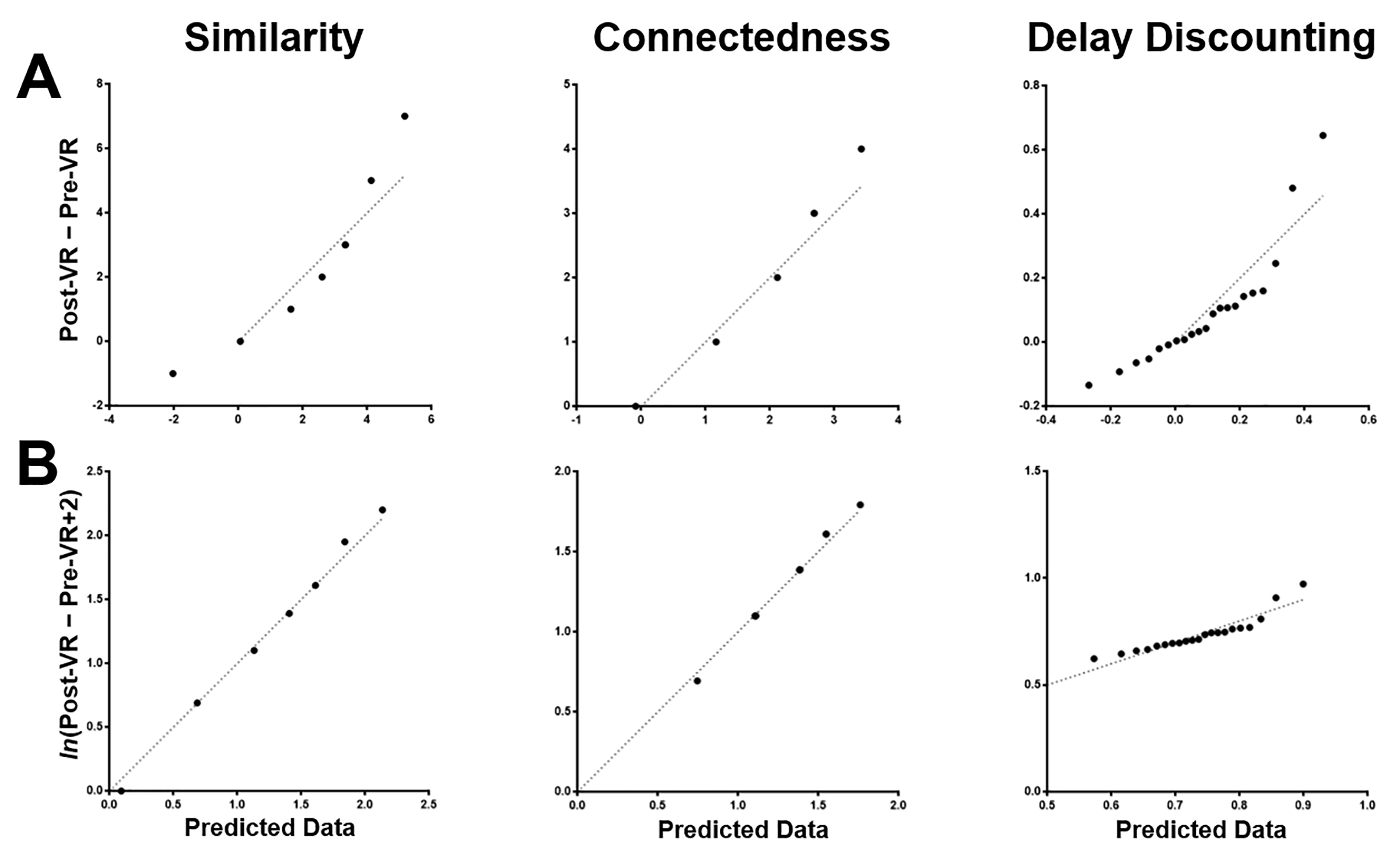

Supplement: Supplementary file 3 — Supplementary file3 Figure S2. Q-Q Plots. (A) Primary outcomes (post-VR minus pre-VR) and (B) natural-log transformed (constant-added positive values) are shown with line of identity (dotted). (TIF 3456 KB) [file 44192_2022_22_MOESM3_ESM.tif]
