# Supplementary material for: Virtual reality intervention effects on future self-continuity and delayed reward preference in substance use disorder recovery: pilot study results
Source: Discov Ment Health. 2022 Sep 15;2(1):19. doi: 10.1007/s44192-022-00022-1 (PMC9477176; doi:10.1007/s44192-022-00022-1)
Supplement: Supplementary file 4 — Supplementary file4 (DOCX 29 KB) [file 44192_2022_22_MOESM4_ESM.docx]

| **Table S1**  **Clinical Characteristics** | |
| --- | --- |
| **Lifetime Diagnosis** | **Total (*N* = 21)**  *n* (%) |
| *Alcohol-Related* |  |
| AUD (DSM-5)^a^ | 19 (90.5) |
| AUD exclusive^b^ | 2 (9.5) |
| *Illicit Substance-Related* |  |
| Illicit Poly Substance Use Disorder^c^ | 17 (81) |
| Stimulants | 13 (61.9) |
| Cannabis | 12 (57.1) |
| Opioids | 8 (38.1) |
| Sedatives | 6 (28.6) |
| Hallucinogens | 3 (14.3) |
| Inhalants^d^ | 3 (14.3) |
| *Self-reported Past Clinical Diagnosis* |  |
| Major depression^e^ | 14 (66.7) |
| Bipolar^f^ | 4 (19.0) |
| Anxiety^g^ | 13 (61.9) |
| ADHD | 3 (14.3) |
| PTSD | 1 (4.8) |

^a^Assessed with SCID-RV v1.0.0

^b^Meeting diagnostic criteria for AUD only (lifetime), i.e., illicit drug use 6 or fewer times in any year and lacking SUD diagnosis.

^c^Greater than one lifetime SUD met, excluding AUD.

^d^Including aerosol propellants, nitrous oxide, and nitrite inhalants.

^e^Including unipolar depression.

^f^Including one participant with comorbid unipolar depression.

^g^Including generalized anxiety and panic disorder.

ADHD, Attention-Deficit / Hyperactivity Disorder; PTSD, Post-Traumatic Stress Disorder

| **Table S2**  **VR Questionnaire and Ratings** | | | |
| --- | --- | --- | --- |
| **Concept** | **Statement** | Mean (SD) | |
| *Presence*^a^ |  | |  |
| Plausibility | “How much did the setting feel like the real world?” | | 4.57 (1.21) |
| Place illusion | “How much did you feel like you were in the future?” | | 4.86 (1.62) |
| Copresence | “How much did you feel like you were really with an actual person?  “How much did Future Self seem like a real person?” | | 4.43 (1.69)  4.81 (1.69) |
| *Tolerability*^b^ |  | |  |
| VR Sickness | “Right now, I feel bad overall” | | 1.19 (0.51) |
|  | “Right now, I have a headache” | | 1.05 (0.22) |
|  | “Right now, my eyes hurt” | | 1.05 (0.22) |
|  | “Right now, I am having trouble seeing”  “Right now, I am sick to my stomach”  “Right now, I am having trouble concentrating” | | 1.05 (0.22)  1.10 (0.30)  1.10 (0.30) |
|  | “Right now, I am dizzy” | | 1.05 (0.22) |
| *Enjoyability*^c^ |  | |  |
| Liking | “I like using the virtual reality headset” | | 6.57 (0.68) |
| Comfort | “The headset is comfortable” | | 6.19 (0.81) |
| Headset weight | “The headset is too heavy” | | 1.81 (0.93) |

^a^1-7 scale; from “Not at all” to “Very Much”

^b^1-4 scale; from “None” to “Severe”

^c^1-7 scale; from “Strongly Disagree” to “Strongly Agree”

| **Table S3**  **Emotion States** | | |  | |  |  |
| --- | --- | --- | --- | --- | --- | --- |
| **Emotions** | **Items** | **Pre VR**  Mean (SD) | | **Post VR**  Mean (SD) | |  |
| *Positive* |  |  | |  | |  |
|  | Happy | 5.30 (1.08) | | 5.05 (1.60) | |  |
|  | Optimistic | 5.35 (1.04) | | 5.48 (1.72) | |  |
|  | Excited | 5.15 (1.69) | | 5.05 (1.60) | |  |
|  | Joyful | 4.70 (1.72) | | 4.67 (1.77) | |  |
|  | Hopeful | 5.05 (1.67) | | 5.38 (1.60) | |  |
|  | Proud | 4.95 (1.73) | | 4.95 (1.88) | |  |
|  | Curious | 6.05 (1.47) | | 5.33 (1.53) | |  |
|  | Engaged | 5.75 (1.12) | | 5.81 (0.98) | |  |
| *Negative* |  |  | |  | |  |
|  | Angry | 1.00 (0.00) | | 1.19 (0.87) | |  |
|  | Anxious | 2.45 (1.36) | | 2.38 (1.60) | |  |
|  | Frustrated | 1.05 (0.22) | | 1.48 (1.33) | |  |
|  | Fearful | 1.40 (0.82) | | 1.76 (1.00) | |  |
|  | Sad | 1.30 (0.73) | | 2.00 (1.38) | |  |
|  | Confused | 1.80 (1.32) | | 1.62 (1.43) | |  |
|  | Bored | 1.85 (1.09) | | 1.38 (0.67) | |  |
|  | Distracted | 2.00 (1.45) | | 1.62 (0.97) | |  |
| All items were rated on a 1-7 Likert-style scale from “Not at all” to “Extremely.” | | | | | | |

| **Table S4**  **Non-Directive Interview: VR Intervention Subjective Feedback** | | | |  |
| --- | --- | --- | --- | --- |
| **Participant** | **Subjective Feedback** | **Future Self-Continuity^a^ Change Score**  **(pre- vs. post-VR)** | | |
| 0027 | “It was a really powerful experience… seeing myself older with a dirty shirt… I don't want to be that guy in 15 years. I would love to have a copy of the video of my selves talking to me. I want to be able to hold onto this. If I am feeling weak in my recovery, I want to be able to just pull it up on my phone and watch it. Having a virtual representation of the future is helpful because in recovery, we live in the present, so we don't know what the future holds—my life is the same now as it was then, and I don't have much to show for it. If I stay on the path, I can see the life I envisioned for myself. ” | | 3 |  |
| 0026 | “Putting different futures together, you don't really visually [see that]. How many people tell you over and over this and this and this can happen. Even though it wasn't hyper realistic it was still something to think about.” | | 3 |  |
| 0040 | “Hearing them [Avatars] say, the one that chose to continue drinking and the one that chose not to; just hearing one say this is what life is like and the other one saying this is what life is like—so I could relate to that.” | | 2 |  |
| 0071 | “If I could talk to myself, this would be something that I could say to myself. This experience helped to reinforce what I think about in recovery. Seeing it in a virtual perspective just reaffirms it.” | | 2 |  |
| 0080 | “It was a wonderful experience… Once I seen myself in the mirror, I was like whoa… that's me! Having myself speak to myself about the choices I have made, and seeing the bad future and good future, was life changing.” | | 9 |  |
| 0093 | “I was shocked… in the active addiction future, seeing myself (Avatar) twitch and move around a lot it made me realize that was me, that's what I looked like. The reality of the situation… that could be me in 15 years if I don't do what I need to do. Hearing it from external forces—we have to rely on others to tell us we're worth recovery, but what if they change their mind? In recovery, we have to constantly work on self-love and self-respect... hearing it from the avatar reaffirms the self-work I've put in.” | | 2 |  |

^a^Sum of future self-similarity and future self-connectedness
